# Significantly Enhanced Crystallization of Poly(ethylene succinate-*co*-1,2-propylene succinate) by Cellulose Nanocrystals as an Efficient Nucleating Agent

**DOI:** 10.3390/polym14020224

**Published:** 2022-01-06

**Authors:** Siyu Pan, Zhiguo Jiang, Zhaobin Qiu

**Affiliations:** State Key Laboratory of Chemical Resource Engineering, Beijing University of Chemical Technology, Beijing 100029, China; 2020200319@grad.buct.edu.cn

**Keywords:** poly(ethylene succinate-*co*-1,2-propylene succinate), cellulose nanocrystals, crystallization

## Abstract

Poly(ethylene succinate-*co*-1,2-propylene succinate) (PEPS) is a novel aliphatic biodegradable polyester with good mechanical properties. Due to the presence of methyl as a side group, the crystallization rate of PEPS is remarkably slower than that of the poly(ethylene succinate) homopolymer. To promote the potential application of PEPS, the effect of cellulose nanocrystals (CNC) on the crystallization behavior, crystalline morphology, and crystal structure of PEPS was investigated in this research with the aim of increasing the crystallization rate. CNC enhanced both the melt crystallization behavior of PEPS during the cooling process and the overall crystallization rate during the isothermal crystallization process. The crystallization rate of PEPS became faster with an increase in CNC content. The crystalline morphology study directly confirmed the heterogeneous nucleating agent role of CNC. The crystal structure of PEPS remained unchanged in the composites. On the basis of the interfacial energy, the nucleation mechanism of PEPS in the composites was further discussed by taking into consideration the induction of CNC.

## 1. Introduction

Due to the growing concerns regarding both fossil resource deficiency and environment protection, bio-based and biodegradable aliphatic polyesters have been the research focus, from a sustainable viewpoint, ofboth the academic and industrial fields in recent decades [1,2,3,4,5,6]. Poly(ethylene succinate) (PES) is a typical member of bio-based and biodegradable aliphatic polyesters, as the monomers to synthesize PES, i.e., ethylene glycol and succinic acid, may be derived from either fossil resource or bio-based resources. It shows a melting point (*T*_m_) of approximately 103 °C with a glass transition temperature (*T*_g_) of −11 °C; in addition, the tensile mechanical property of PES is comparable to those of low-density polyethylene and polypropylene [7,8,9,10,11,12,13]. For instance, it displays a tensile modulus (*E*_t_) of 409 ± 13 MPa, a tensile strength (σ) of 23.4 ± 1.5 MPa, and an elongation at break (ε) of 285 ± 30% [14]. To modify the physical properties and meet various practical application requirements, some PES based copolymers have been synthesized through the copolymerization method by introducing a new linear diol or diacid monomer during the polymerization process of PES [15,16,17,18,19,20]. With respect to PES, these copolymers usually display lower *T*_m_ values and better toughness with higher ε values [15,16,17,18,19,20]. In addition, some novel PES based copolymers with different lengths of side groups have also been synthesized and studied in the literature by the use of some 1,2-diols, such as 1,2-propanediol, 1,2-hexanediol, and 1,2-decanediol, as comonomers [14,21,22,23,24]. These novel PES-based copolymers show the same main chain structures as PES, except that some side groups (such as methyl, butyl, and octyl) are randomly linked to the polymer main chain. In a previous study, the effect of different lengths of side groups on the thermal, crystallization, and mechanical properties of PES were systematically investigated [24]. Among these copolymers, poly(ethylene succinate-*co*-1,2-propylene succinate) (PEPS) is of great importance and interest, as the presence of a small amount of the simplest C−H side group of methyl (−CH_3_) can lead to a remarkable change of the physical properties of PES [14,24]. For instance, the storage modulus, loss modulus, and complex viscosity of PEPS were significantly higher than those of PES; even the 1,2-propylene succinate (PS) unit was only approximately 5 mol%. In addition, the tensile mechanical property of PEPS was superior to that of PES. For instance, PEPS with roughly 5 mol% of PS unit showed a higher σ of 40.9 ± 4.1 MPa and a higher ε of 755 ± 91%, while the σ and ε values of PES were only 23.4 ± 1.5 MPa and 285 ± 30%, respectively [14]. Although PEPS showed better rheological and tensile mechanical properties than PES, the crystallization rate of PEPS became slower due to the random copolymer feature. From a practical application viewpoint, the crystallization rate of PEPS should be remarkably enhanced. So far, the use of heterogeneous nucleating agents has been regarded as the most efficient method to accelerate the crystallization of polymer materials because it may both provide sufficient active nucleating sites and reduce the nucleation activation energy barrier of polymer crystallization [7,8,9,10,11,12,13,23].

Among the widely used nucleating agents, cellulose nanocrystals (CNC) are of particular interest. The characteristics of CNC are as follows: bio-based, biodegradable, highly crystalline, rod-like, high aspect ratio, and superior mechanical property; in addition, the hydroxyl groups on the surface also provide the possibility of chemical modification to increase the solubility in organic solvent and the compatibility with polymer matrix [25,26,27,28]. So far, some CNC nucleated biodegradable polymers have been reported in the literature, such as poly(ε-caprolactone) (PCL), poly(L-lactic acid) (PLLA), PES, poly(butylene succinate) (PBS), poly(hexamethylene succinate) (PHS), poly(butylene succinate-*co*-butylene adipate) (PBSA), and poly(ethylene adipate) (PEA) [29,30,31,32,33,34,35,36,37,38,39,40].

In this research, we prepared low contents of CNC nucleated PEPS composites and extensively studied the effect of CNC as a heterogeneous nucleating agent on the crystallization of PEPS. CNC could induce the melt crystallization of PEPS at a relatively fast cooling rate of 20 °C/min; moreover, CNC remarkably shortened the crystallization time and crystallization half-time of PEPS during the isothermal crystallization indicating the efficient nucleating agent effect. The significance of this study is summarized as follows. On the one hand, PEPS/CNC composites, both of which were biodegradable, were prepared and studied for the first time. On the other hand, CNC significantly accelerated the crystallization of PEPS under different crystallization conditions; moreover, we further reasonably discussed the nucleation mechanism of PEPS induced by CNC on the basis of the interfacial energy. Therefore, this research is important and interesting in the fields of both polymer crystallization and biodegradable polymer composites.

## 2. Experimental Section

### 2.1. Materials

PEPS (*M*_n_ = 5.3 × 10^4^ g/mol, PDI = 1.89, and PS content = 4.1 mol%) was synthesized via a two-stage melt polycondensation reaction in our laboratory [14]. CNC (with an average diameter of 5~20 nm and length of 50~200 nm) was produced by Shanghai ScienceK Nanotechnology Co., Ltd. *N,N*-dimethylformamide (DMF) (purity = 99.5%) was bought from Tianjin Damao Chemical Reagent Factory, China.

**Figure 1:**
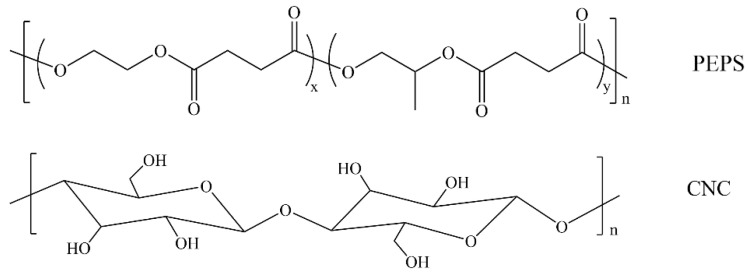
Chemical structures of PEPS and CNC.

The chemical structures of PEPS and CNC are illustrated in Figure 1.

### 2.2. Preparation of PEPS/CNC Composites

Three PEPS/CNC composites, i.e., PEPS/CNC0.25, PEPS/CNC0.5, and PEPS/CNC1, were prepared in this research, with the number being the wt% of CNC. The preparation procedure of PEPS/CNC1 was simply described as follows. First, PEPS (2.97 g) was dissolved into DMF (35 mL) at 40 °C for 2.5 h, and CNC (30 mg) was dispersed into DMF (15 mL) after a sonication process of 2.5 h. Second, the PEPS solution and the CNC dispersion were mixed together at 40 °C for 4.5 h. Third, the film was obtained after evaporating DMF at 40 °C for one night in a fume hood and for 7 days in a vacuum oven. Similarly, PEPS/CNC0.25 and PEPS/CNC0.5 were also prepared.

### 2.3. Characterizations

Thermogravimetric analysis (TGA) was performed on a TA instrument Q50 to study the thermal stability of PEPS and PEPS/CNC composites under nitrogen atmosphere at a heating rate of 20 °C/min.

The crystallization behavior of PEPS and PEPS/CNC composites was investigated with a TA Q100 differential scanning calorimeter (DSC) under a nitrogen atmosphere. The weight of each sample was approximately 4~5 mg. For each test, the thermal history of a fresh sample was first eliminated by heating at 40 °C/min to 130 °C (almost 40 °C above the *T*_m_ of 93.9 °C for PEPS) and holding there for 3 min. The nonisothermal melt crystallization behavior of PEPS and PEPS/CNC composites was studied at a cooling rate of 20 °C/min after the elimination of the previous thermal history of the samples. In the case of the isothermal melt crystallization kinetics study, the sample was isothermally crystallized at the chosen crystallization temperature (*T*_c_) for sufficient time after cooling from the crystal-free melt at 60 °C/min after the elimination of previous thermal history. In this research, the crystallization was studied in a *T*_c_ range from 63 to 71 °C.

A polarized optical microscope (POM) (Olympus BX51) equipped with a hot stage (Linkam THMS 600) was used to observe the spherulitic morphology of PEPS and PEPS/CNC composites.

A Rigaku Ultima IV X-ray diffractometer was operated at 40 kV and 200 mA to study the crystal structures of PEPS and PEPS/CNC composites. The wide-angle X-ray diffraction (WAXD) experiments were performed with a rate of 5°/min at ambient temperature in a 2θ range of 5° to 40°. The samples for the WAXD measurement underwent an isothermal crystallization at 63 °C for 8 h in an oven.

## 3. Results and Discussion

**Figure 2:**
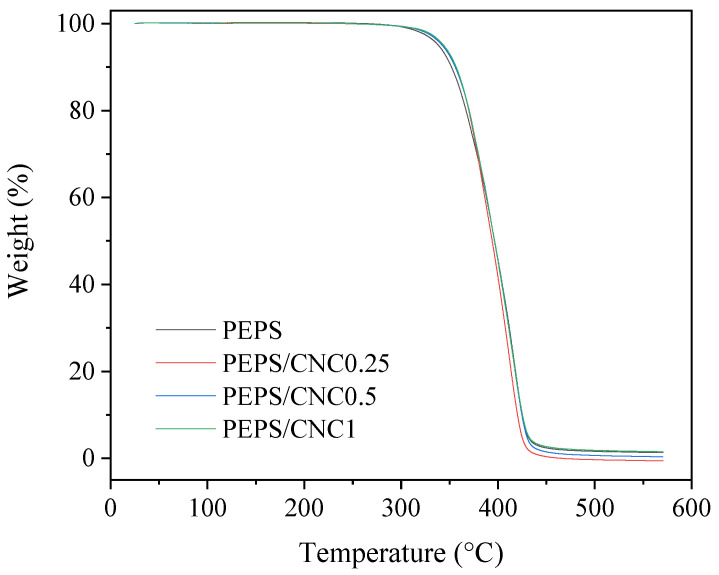
TGA curves of PEPS and PEPS/CNC composites.

Thermal decomposition temperature (*T*_d_) is an important physical parameter from the viewpoints of both polymer processing and the long time use at elevated temperatures. The influence of CNC on the thermal stability of PEPS was first explored with TGA at a heating rate of 20 °C/min under a nitrogen atmosphere. Figure 2 demonstrates the TGA curves of PEPS and PEPS/CNC composites, from which one-step thermal decomposition behavior was found for all samples, irrespective of CNC content. The *T*_d_ values, corresponding to 5 wt% weight losses, were read from Figure 2, which increased slightly from 338.3 °C for PEPS to about 343.7 °C for the composites. The slight increase in *T*_d_ arose from the presence of CNC, which played a role in physical barriers and hindered the heat transfer and permeation of combustion gas in the PEPS matrix [35].

**Figure 3:**
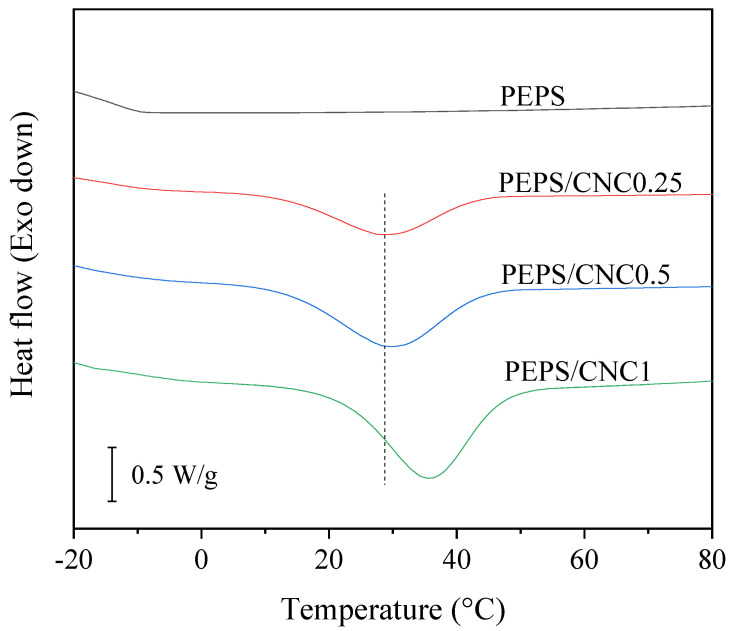
Melt crystallization behavior of PEPS and PEPS/CNC composites at 20 °C/min.

Although PEPS has the same main chain structure as PES, the presence of methyl as a side group destroys the regularity of the main chain structure. As a result, the crystallizability of PEPS is remarkably weaker than that of the PES homopolymer. The DSC cooling traces are depicted in Figure 3 for PEPS and PEPS/CNC composites, which were nonisothermally crystallized at a fast cooling rate of 20 °C/min from the crystal-free melt. Under this crystallization condition, PEPS did not crystallize, showing no crystallization exothermic peak in Figure 3. On the contrary, regardless of CNC content, the three PEPS/CNC composites showed obvious crystallization exothermic peaks during the crystallization process, indicating that CNC played an outstanding nucleating agent role and enhanced the crystallization of PEPS. From Figure 3, the melt crystallization temperature (*T*_mc_) was determined. The *T*_mc_ values gradually increased from 28.3 °C for PEPS/CNC0.25 to 29.7 °C for PEPS/CNC0.5 and 35.5 °C for PEPS/CNC1, respectively. Similarly, the melt crystallization enthalpy (Δ*H*_mc_) remarkably increased from 21.9 J/g for PEPS/CNC0.25 to 47.1 and 54.6 J/g for PEPS/CNC0.5 and PEPS/CNC1, respectively. By using the equilibrium heat of fusion of PES (180 J/g) [10], the absolute degree of crystallinity values of the three composites was approximately calculated to be 12.2%, 26.2% and 30.3%, respectively. The obvious increase in both *T*_mc_ and Δ*H*_mc_ revealed that CNC remarkably enhanced the melt crystallization of PEPS even at a relatively fast cooling rate of 20 °C/min as an efficient nucleating agent.

**Figure 4:**
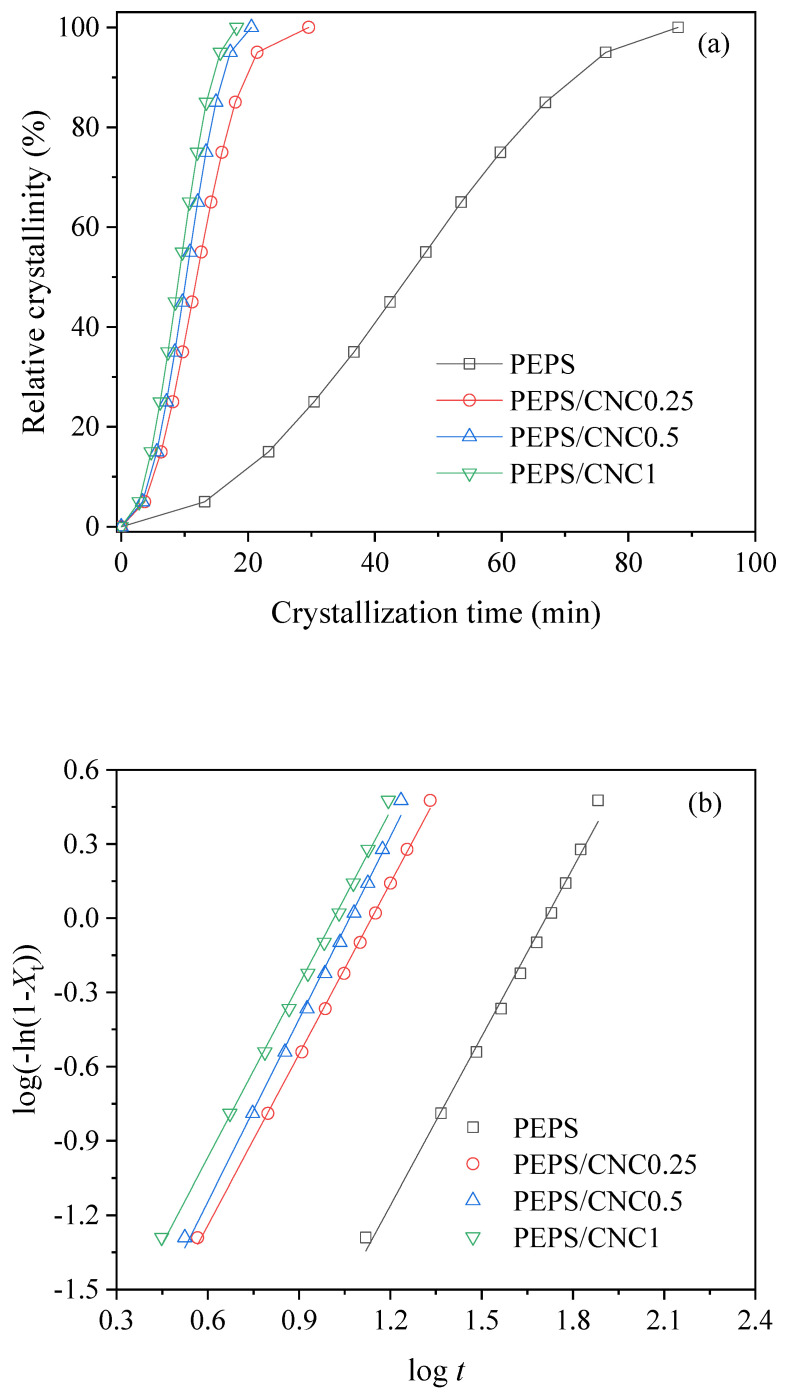
(**a**) Plots of relative crystallinity with crystallization time and (**b**) Avrami plots for PEPS and PEPS/CNC composites at 71 °C.

The effect of CNC on the isothermal melt crystallization kinetics of PEPS was further investigated with DSC in this research. Figure 4a show the plots of relative crystallinity versus crystallization time of PEPS and PEPS/CNC composites at a *T*_c_ of 71 °C. Due to the small degree of supercooling, PEPS crystallized slowly and required 87.8 min to complete the crystallization, while the total crystallization time for PEPS remarkably became shorter in the composites. For instance, PEPS/CNC0.25 needed 29.6 min to finish the crystallization, while PEPS/CNC1 even only required 18.2 min at the same *T*_c_, suggesting that the higher the CNC content, the shorter the crystallization time. The significantly short crystallization time of PEPS/CNC composites indicated that the isothermal melt crystallization of PEPS was also promoted by CNC as an effective nucleating agent.

The well-known Avrami equation was utilized to analyze the isothermal crystallization kinetics of PEPS and PEPS/CNC composites. Relative crystallinity shows a relationship with crystallization time as follows:

1 − *X*_t_ = exp(−*kt^n^*) (1)

where *X*_t_ is the relative crystallinity at crystallization time (*t*), *k* is the crystallization rate constant, and *n* is the Avrami exponent [41,42,43,44,45,46]. Figure 4b depict the related Avrami plots at 71 °C, showing almost parallel fitting lines for PEPS and PEPS/CNC composites, from which the *n* and *k* values were obtained. PEPS and PEPS/CNC showed similar results when they were crystallized at other *T*_c_ values. For simplicity, they are not shown here. Table 1 summarize the related isothermal crystallization kinetics parameters for PEPS and PEPS/CNC composites in the investigated *T*_c_ range.

From Table 1, the *n* values were between 2 and 3 for PEPS and PEPS/CNC composites, indicating that CNC did not change the crystallization mechanism of PEPS within the investigated *T*_c_ range. For both PEPS and PEPS/CNC composites, increasing *T*_c_ gradually decreased the *k* values, suggesting that the crystallization rate became slower with the increase of *T*_c_. The *k* values gradually increased with increasing CNC content at the same *T*_c_, indicating that the crystallization rate of PEPS became faster due to the nucleating agent role of CNC. It should be emphasized that the unit of *k* values was min^−*n*^, while the *n* values varied slightly with *T*_c_ and CNC content for PEPS and PEPS/CNC composites. Therefore, for an accurate comparison of the crystallization rate in this research, crystallization half-time (*t*_0.5_) with the same unit (min) was used. Through the Avrami equation, *t*_0.5_ was calculated as follows using the *n* and *k* values listed in Table 1:

(2) $$t_{0.5}={\left(\frac{\ln2}{k}\right)}^{1/n}$$

The acquired *t*_0.5_ values are summarized in Table 1, too. As displayed in Table 1, *t*_0.5_ increased with *T*_c_ for both PEPS and PEPS/CNC composites, indicating a slower crystallization rate; moreover, *t*_0.5_ of PEPS/CNC composites gradually decreased with increasing CNC content, suggesting the increased crystallization rate. In addition, crystallization half-time (*t*_1/2_) could also be directly read from the plots of relative crystallinity versus crystallization time (Figure 4a), which are also summarized in Table 1 for comparison. It is obvious that the difference between *t*_0.5_ and *t*_1/2_ is very small, indicating that the Avrami equation may well fit the crystallization kinetics of these systems in this research.

**Figure 5:**
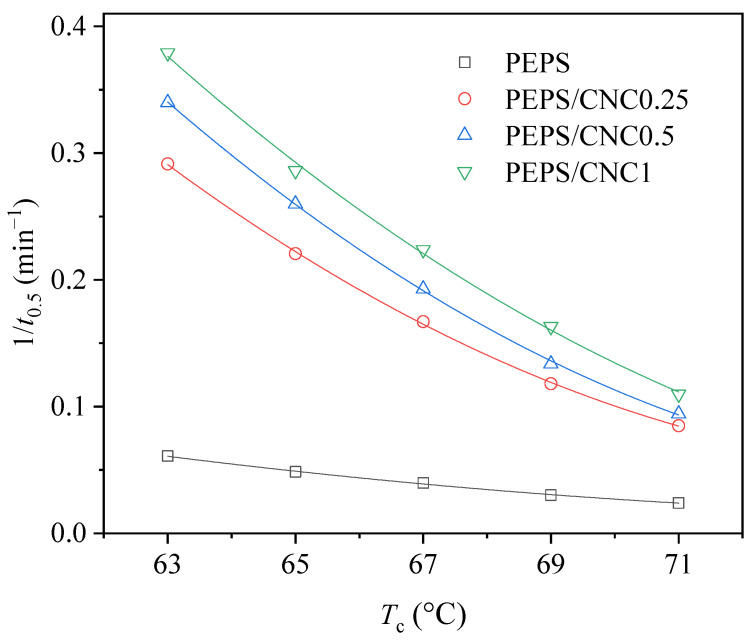
Variation of 1/*t*_0.5_ with *T*_c_ for PEPS and PEPS/CNC composites.

Crystallization rate may easily be described by the reciprocal of *t*_0.5_ (1/*t*_0.5_) with the same unit (min^−1^) in polymer crystallization. The greater the 1/*t*_0.5_, the faster the crystallization rate. To show the effect of *T*_c_ and CNC content on the crystallization rate more clearly, Figure 5 demonstrate the variation of 1/*t*_0.5_ with *T*_c_ for all samples. On the one hand, 1/*t*_0.5_ increased with decreasing *T*_c_ for each sample, suggesting the faster crystallization rate due to the larger degree of supercooling. On the other hand, the 1/*t*_0.5_ values of PEPS/CNC composites were remarkably greater than that of PEPS at the same *T*_c_ and became gradually greater with increasing CNC content. The above result revealed that CNC, as an effective heterogeneous nucleating agent, obviously enhanced the crystallization rate of PEPS

**Figure 6:**
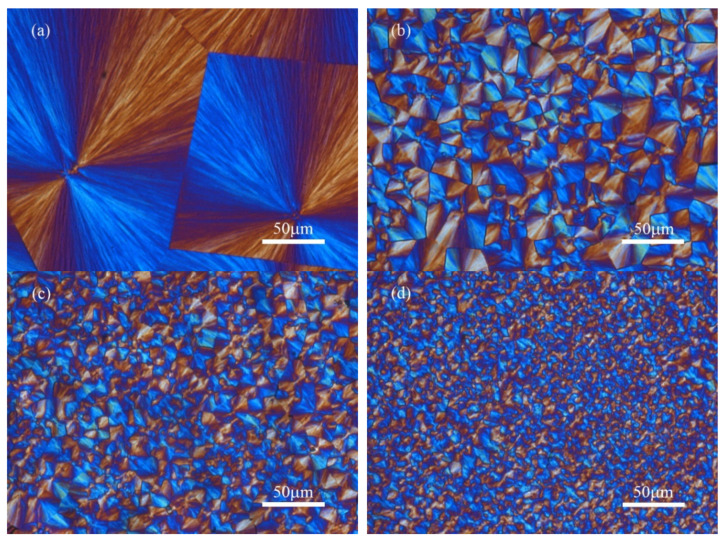
POM micrographs for (**a**) PEPS, (**b**) PEPS/CNC0.25, (**c**) PEPS/CNC0.5, and (**d**) PEPS/CNC1 at 63 °C.

As mentioned above, CNC remarkably enhanced the crystallization behavior of PEPS under different crystallization conditions, indicating the nucleating agent role of CNC. In this section, the spherulitic morphology of PEPS and PEPS/CNC composites was directly observed with a hot-stage POM. Figure 6 illustrate the POM micrographs after PEPS and PEPS finished the crystallization at 63 °C and filled the entire space. In Figure 6a, several relatively large negative spherulites were observed for the unmodified PEPS due to the small degree of supercooling. In the case of the composites, as shown in the rest of Figure 6, the negative PEPS spherulites were still found. CNC significantly increased the number of PEPS spherulites and accordingly reduced the size of spherulites, suggesting the efficient nucleating agent role. Furthermore, the higher the CNC content, the stronger the nucleating agent effect. Despite the variation of CNC content, the spherulitic growth rates of PEPS and PEPS/CNC composites were about 2.60 μm/min. In other words, CNC only increased the nucleation density of PEPS spherulites and did not influence the growth rate. In brief, the crystalline morphology study directly confirmed that CNC enhanced the crystallization of PEPS as an outstanding heterogeneous nucleating agent by increasing the nucleation density of PEPS spherulites in the composites.

**Figure 7:**
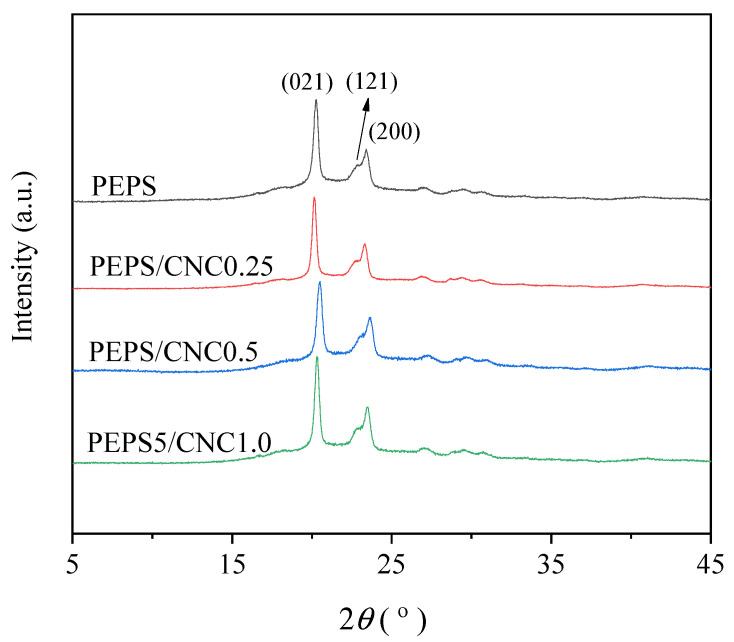
WAXD patterns of PEPS and PEPS/CNC composites.

Figure 7 displays the WAXD profiles of PEPS and PEPS/CNC composites after crystallizing at 63 °C for 8 h. PEPS showed the same crystal structure as the PES homopolymer, presenting three main diffraction peaks at 2*θ* of 20.3°, 22.9°, and 23.5°, which were attributed to (021), (121), and (200) planes, respectively [14,47]. In the case of the composites, they demonstrated similar WAXD profiles as PEPS, despite CNC content, suggesting that both the composites and PEPS shared the same crystal structure. From the WAXD profiles of Figure 7, the crystallinity values were calculated to be 54 ± 2% for PEPS and PEPS/CNC composites by separating the crystalline region and amorphous region of the WAXD profile in Figure 7. In brief, the crystal structure and crystallinity of PEPS remained unchanged in the composites despite the presence of CNC.

From the above studies, CNC enhanced the crystallization of PEPS during both nonisothermal and isothermal melt crystallization processes. As an effective heterogeneous nucleating agent, CNC only promoted the nucleation density and hardly influenced the spherulitic growth rate of PEPS spherulites. It is interesting to discuss the effect of CNC on the enhanced nucleation density of PEPS spherulites from the viewpoint of the interfacial energy (γ_12_) between CNC and PEPS. In the literature, the γ_12_ value between filler and polymer matrix may be calculated through the well-known harmonic mean equation or the geometric mean equation described as follows [48]:

(3) $$\gamma_{12}=\gamma_{1}+\gamma_{2}-4\left(\frac{\gamma_{1}^{d}\gamma_{2}^{d}}{\gamma_{1}^{d}+\gamma_{2}^{d}}+\frac{\gamma_{1}^{p}\gamma_{2}^{p}}{\gamma_{1}^{p}+\gamma_{2}^{p}}\right)$$

(4) $$\gamma_{12}=\gamma_{1}+\gamma_{2}-2\left(\sqrt{\gamma_{1}^{d}\gamma_{2}^{d}}+\sqrt{\gamma_{1}^{p}\gamma_{2}^{p}}\right)$$

where γ_1_ and γ_2_ are the surface energy of the two components, i.e., CNC and PEPS, respectively, in this work; *γ^d^* and *γ^p^* are dispersive components and the polar component of each component, respectively. As PEPS is a new biodegradable polymer, we calculated the surface energy, dispersive component, and polar component through the contact angle measurements using water and ethylene glycol as the solvent according to the classical method proposed by Owens and Wendt [49]. For simplicity, the details are not described here. The calculated values of PEPS are listed in Table 2. In addition, the relevant values of CNC reported in the literature are also listed in Table 2 for the calculation of γ_12_ [50].

On the basis of the data listed in Table 2, the γ_12_ values were calculated to be 11.96 or 6.28 mJ/m^2^ through Equation (3) or (4), respectively. The relatively small γ_12_ value indicated a good interfacial affinity between PEPS and CNC. Consequently, CNC may not only provide sufficient active heterogeneous nucleation sites for PEPS chain to nucleate and grow on the surface but also lower the nucleation barrier, thereby increasing the nucleation rate and further overall crystallization rate.

## 4. Conclusions

In this research, low contents of CNC nucleated PEPS composites were successfully prepared through a solution and casting method with the aim of increasing the crystallization rate of PEPS for its potential practical application. CNC slightly increased the thermal stability of PEPS. Under both nonisothermal and isothermal melt crystallization conditions, CNC promoted the crystallization of PEPS. At a fast cooling rate of 20 °C/min, PEPS could not crystallize during the crystallization process, showing no crystallization exotherm, while PEPS/CNC composites showed well-defined crystallization exotherms. For instance, 1 wt% of CNC nucleated PEPS showed a melt crystallization temperature of 35.5 °C with a melt crystallization enthalpy of 54.6 J/g, indicating the efficient nucleating agent effect of CNC. Due to the small degree of supercooling, the crystallization half-time gradually became shorter for the unmodified and nucleated PEPS at higher crystallization temperatures. At the same crystallization temperature, CNC remarkably shortened the crystallization half-time and increased the crystallization rate of PEPS; furthermore, the higher the CNC content, the faster the crystallization rate. For example, 1 wt% of CNC significantly decreased the crystallization half-time of PEPS from 16.4 to 2.8 min. The crystalline morphology and crystal structure studies indicated that CNC did not change the growth rate and crystal structure of PEPS but increased the nucleation density of PEPS spherulites. On the basis of the harmonic mean equation or the geometric mean equation, the interfacial energy of PEPS-CNC was determined to be 11.96 or 6.28 mJ/m^2^, respectively. The small interfacial energy proved the good affinity between the PEPS chain and CNC surface. In other words, the PEPS chain should be easier to attach, nucleate, and further grow on the surface of CNC; therefore, CNC, as an efficient, biodegradable heterogeneous nucleating agent, significantly enhanced the crystallization of PEPS.

## Figures and Tables

**Table 1:** Isothermal crystallization kinetics parameters for PEPS and PEPS/CNC composites.

Samples	*T*_c_ (°C)	*n*	*k* (min^−*n*^)	*t*_0.5_ (min)	*t*_1/2_ (min)
PEPS	63	2.1	1.94 × 10^−3^	16.4	16.0
	65	2.2	8.90 × 10^−4^	20.6	20.0
	67	2.2	5.71 × 10^−4^	25.2	26.7
	69	2.2	3.10 × 10^−4^	33.3	32.1
	71	2.3	1.30 × 10^−4^	41.8	45.1
PEPS/CNC0.25	63	2.6	2.81 × 10^−2^	3.4	3.5
	65	2.5	2.30 × 10^−2^	3.9	4.2
	67	2.5	7.90 × 10^−3^	6.0	6.2
	69	2.4	6.37 × 10^−3^	7.1	8.4
	71	2.3	2.38 × 10^−3^	11.8	11.8
PEPS/CNC0.5	63	2.6	4.19 × 10^−2^	2.9	2.9
	65	2.7	1.82 × 10^−2^	3.8	3.9
	67	2.6	8.43 × 10^−3^	5.5	5.3
	69	2.4	7.30 × 10^−3^	6.7	7.1
	71	2.4	2.39 × 10^−3^	10.6	10.2
PEPS/CNC1	63	2.8	3.72 × 10^−2^	2.8	2.7
	65	2.6	2.68 × 10^−2^	3.5	3.5
	67	2.4	1.90 × 10^−2^	4.5	4.6
	69	2.3	1.52 × 10^−2^	5.3	5.9
	71	2.3	4.30 × 10^−3^	9.1	9.0

**Table 2:** Surface energy data of PEPS and CNC.

Samples	γ (mN/m)	*γ^d^* (mN/m)	*γ^p^* (mN/m)
PEPS	31.1	14.5	16.7
CNC	60.7	39.4	21.3

## Data Availability

The data presented in this study are available on request from the corresponding author.
